# Extrauterine growth restriction in very-low-birthweight infants: prevalence and concordance according to Fenton, Olsen, and INTERGROWTH-21st growth charts in a multicenter Spanish cohort

**DOI:** 10.1007/s00431-024-05673-6

**Published:** 2024-07-03

**Authors:** Clara González López, Gonzalo Solís Sánchez, Belén Fernández Colomer, Laura Mantecón Fernández, Sonia Lareu Vidal, Rosa Patricia Arias Llorente, Aleida Ibáñez Fernández, Lara Gloria González García, Marta Suárez Rodríguez

**Affiliations:** 1 Servicio de Neonatología, Área de Gestión Clínica de La Infancia y Adolescencia, Hospital Universitario Central de Asturias, Oviedo, Spain; 2 Instituto Investigación Sanitaria Principado de Asturias, ISPA, Oviedo, Spain; 3 Departamento de Medicina, Universidad de Oviedo, Oviedo, Spain; 4 Servicio de Pediatría, Hospital Universitario de Cabueñes, Gijón, Spain

**Keywords:** Extrauterine growth restriction, Very-low-birthweight infants, Fenton, Olsen, INTERGROWTH-21st

## Abstract

Multiple criteria and growth references have been proposed for extrauterine growth restriction (EUGR). We hypothesized that these may impact the diagnosis of EUGR. The objective was to evaluate the prevalence of EUGR with its different definitions and the concordance according to Fenton, Olsen, and INTERGROWTH-21st in very-low-birthweight (VLBW) infants. This is an observational, retrospective, and multicenter study including VLBW infants from the Spanish SEN1500 Network from 2011 to 2020. Patients with major congenital anomalies, embryopathies, and gestational age less than 24 weeks were excluded. EUGR prevalence was calculated at discharge with cross-sectional, longitudinal, “true” cross-sectional, and “true” longitudinal definitions. Concordance was assessed with Fleiss’ kappa coefficient. 23582 VLBW infants from 77 NICUs were included. In total, 50.4% were men with a median of gestational age of 29 (4) weeks. The prevalence of EUGR (cross-sectional, longitudinal, and “true”) was variable for weight, length, and head circumference. Overall, the prevalence was higher with Fenton and lower with Olsen (cross-sectional and “true” cross-sectional) and INTERGROWTH-21st (longitudinal and “true” longitudinal). Agreement among the charts by weight was good only for cross-sectional EUGR and moderate for longitudinal, “true” cross-sectional, and “true” longitudinal. Concordance was good or very good for EUGR by length and head circumference.

*Conclusions*: The prevalence of EUGR with the most commonly used definitions was variable in the cohort. Agreement among growth charts was moderate for all the definitions of EUGR by weight except cross-sectional and good or very good for length and head circumference. The choice of reference chart can impact the establishment of the diagnosis of EUGR.

**Table:** 

**What is known:** • *EUGR has been defined in the literature and daily practice considering weight, length and head circumference with multiple criteria (cross-sectional, longitudinal, and “true” definition)* • *Different growth charts have been used for EUGR diagnosis*
**What is new:** • *Prevalence of EUGR is variable depending on the definition and growth chart used in our cohort of VLBW infants* • *For the most frequently EUGR criteria used, traditionally considering weight, concordance among Fenton, Olsen and INTERGROWTH-21st growth charts is only moderate for all the definitions of EUGR by weight except cross-sectional definition. Concordance among the charts is good or very good for the different criteria of EUGR by head circumference and length*

**What is known:**

• *EUGR has been defined in the literature and daily practice considering weight, length and head circumference with multiple criteria (cross-sectional, longitudinal, and “true” definition)*

• *Different growth charts have been used for EUGR diagnosis*

**What is new:**

• *Prevalence of EUGR is variable depending on the definition and growth chart used in our cohort of VLBW infants*

• *For the most frequently EUGR criteria used, traditionally considering weight, concordance among Fenton, Olsen and INTERGROWTH-21st growth charts is only moderate for all the definitions of EUGR by weight except cross-sectional definition. Concordance among the charts is good or very good for the different criteria of EUGR by head circumference and length*

## Introduction

Postnatal growth of preterm infants continues to be a challenge in neonatology [1]. Consensus among neonatologists on the ideal extrauterine growth pattern for preterm infants and the optimal practices for monitoring growth in the neonatal intensive care unit (NICU) has not been reached [2].

Extrauterine growth restriction has been described in the literature as a concept to identify inadequate postnatal growth that does not meet the expectations [3]. Despite the frequency of use of the term extrauterine growth restriction both in the clinical practice and literature, there is significant variability on the definitions used and timing for diagnosis for EUGR [4]. Traditionally, EUGR has been described as weight at 36 weeks or discharge below the 10th percentile [5], <  − 2 *Z*-score [6, 7], <  − 1.5 *z*-score [8] or, less frequently, below the 3rd percentile [9] using different growth charts (referred to as cross-sectional definition). An alternate longitudinal definition has been applied to weight loss more than one [9] or two standard [6] deviations from weight at birth. Recently, a new definition has been proposed named as “true” EUGR that would include patients who meet the previously described criteria for EUGR (either cross-sectional definition or longitudinal definition) amongst those not being small for gestational age (SGA) at birth [10, 11]. Several studies have previously described the prevalence of EUGR with the cross-sectional and longitudinal definitions for weight in the neonatal population. However, the literature on prevalence of EUGR with the latest criteria is scarce.

Increasing growth references or standards have been developed and published in the last two decades, with varying methodology, populations, and objectives [12]. Olsen et al. [13] and Fenton et al. [14, 15] growth references are two of the most commonly used in the NICU for preterm infants. Recently, the International Fetal and Newborn Growth Consortium for the 21st Century (INTERGROWTH-21st) published their fetal [16], preterm [17], and newborn [18] growth standards. While Olsen and Fenton are based on transversal anthropometrical data of different populations, the latest was developed based on prospective follow-up of a cohort of newborns with the objective of developing a standard of growth. However, the establishment in preterms of the optimal conditions that would be required for the development of standards of growth can also be challenging. The particularities of the preterm population and the different comorbidities they experience can impact growth and difficult the achievement of a “healthy preterm” [12].

Despite the multiple criteria proposed for EUGR and the frequent use of this term in the literature [4], this term has also raised controversies. A group of experts has highlighted that EUGR could lead to overdiagnosing growth deviations with no clear relation to clinically significant outcomes such as neurodevelopmental [19]. A recent review has also raised the challenges to compare studies of EUGR and neurodevelopment due to the differences in EUGR criteria and neurodevelopmental assessments amongst different papers [20]. Moreover, the previously described group of experts have also suggested revision of the criteria and calculation of the diagnostic accuracy [19]. With this goal, a study from 2024 has evaluated infants born at less than 30 weeks or with a birthweight less than 1500 g from the Preterm Multi-center (PreM) Growth cohort study, describing that despite a significant proportion of preterms plotting low at 36 weeks of postmenstrual age, most of those with adequate nutritional support had experienced catch-up by 3 years of corrected gestational age with a limited ability of growth faltering to predict cognitive scores [21].

The primary objectives of this study are to describe the prevalence of extrauterine growth restriction with the most frequently used definitions (cross-sectional, longitudinal, and “true” EUGR) and to assess the concordance amongst Fenton, Olsen, and INTERGROWTH-21st for those definitions in a multicenter cohort of very-low-birthweight (VLBW) infants in Spain.

## Materials and methods

An observational, retrospective, and multicenter cohort study was conducted including all VLBW newborns from SEN1500 Network. SEN1500 is a national database from the Spanish Society of Neonatology that collects data for preterms with gestational age less than 32 weeks or birthweight less than 1500 g admitted to all voluntarily integrated Spanish level three NICUs.

The study was conducted from January, 1st, 2011 to December, 31st, 2020. All VLBW infants admitted to each participating site in SEN1500 Network during the period of study were eligible for inclusion. Infants with major congenital anomalies, congenital embryopathies with growth impairment (as congenital infection by cytomegalovirus), and gestational age less than 24 weeks (as INTERGROWTH-21st growth chart is not available for those gestational ages) were excluded from the study.

Ethics approval was obtained from “Comité de Ética de la Investigación del Principado de Asturias” with reference number 2022.586. Parents of newborns included in the SEN1500 database provided written consent for the inclusion in the database and its use for clinical studies. Moreover, the authors have no conflict of interests to declare.

Data were collected in accordance to SEN1500 standardized operational definitions, with particular consideration of the criteria proposed by NICHD in 2019 for the diagnosis of bronchopulmonary dysplasia [22].

Percentiles and *Z*-score for weight (W), length (L), and head circumference (HC) of each participant were calculated using the University of Calgary Fenton calculator application [23], Peditool Olsen bulk calculator [24], and the INTERGROWTH-21st calculator application [25, 26], publicly available. For the latest, reference of newborn size for preterm infants was used to evaluate infants at birth while chart of postnatal growth of preterm infant was used to assess postnatal growth. Infants were categorized by weight at birth in small (less than the 10th centile), appropriate (between the 10th and 90th centile) and large for gestational age (more than the 90th centile).

Provided there is no standard definition of EUGR in neonates, we elected to follow the most commonly criteria previously published, defining EUGR as follows: (1) weight at discharge less than 10th centile (cross-sectional definition), (2) decrease of more than 1 SD since birth to the time of discharge home (longitudinal), and (3) “true” EUGR, either cross-sectional or longitudinal EUGR in non-SGA infants at birth. Considering the previously published potential implications of head circumference [27–29] and length [30, 31] in neurodevelopment, we elected to study EUGR with the same previously described definitions for length and head circumference.

All statistical analyses were carried out using R (v 3.4.4, Open-Source International Collaborative), R Studio (v 1.1.463, Open-Source International Collaborative) and SPSS (V27). Basic descriptive statistics were used to characterize data. As the majority of the quantitative variables were not normally distributed, median and interquartile range were used to describe quantitative variables, and percentages were used for the qualitative variables. The primary outcome of interest (concordance between Fenton, Olsen, and INTERGROWTH-21st growth references) was assessed with Fleiss’ kappa coefficient.

## Results

### Study population

**Fig. 1:**
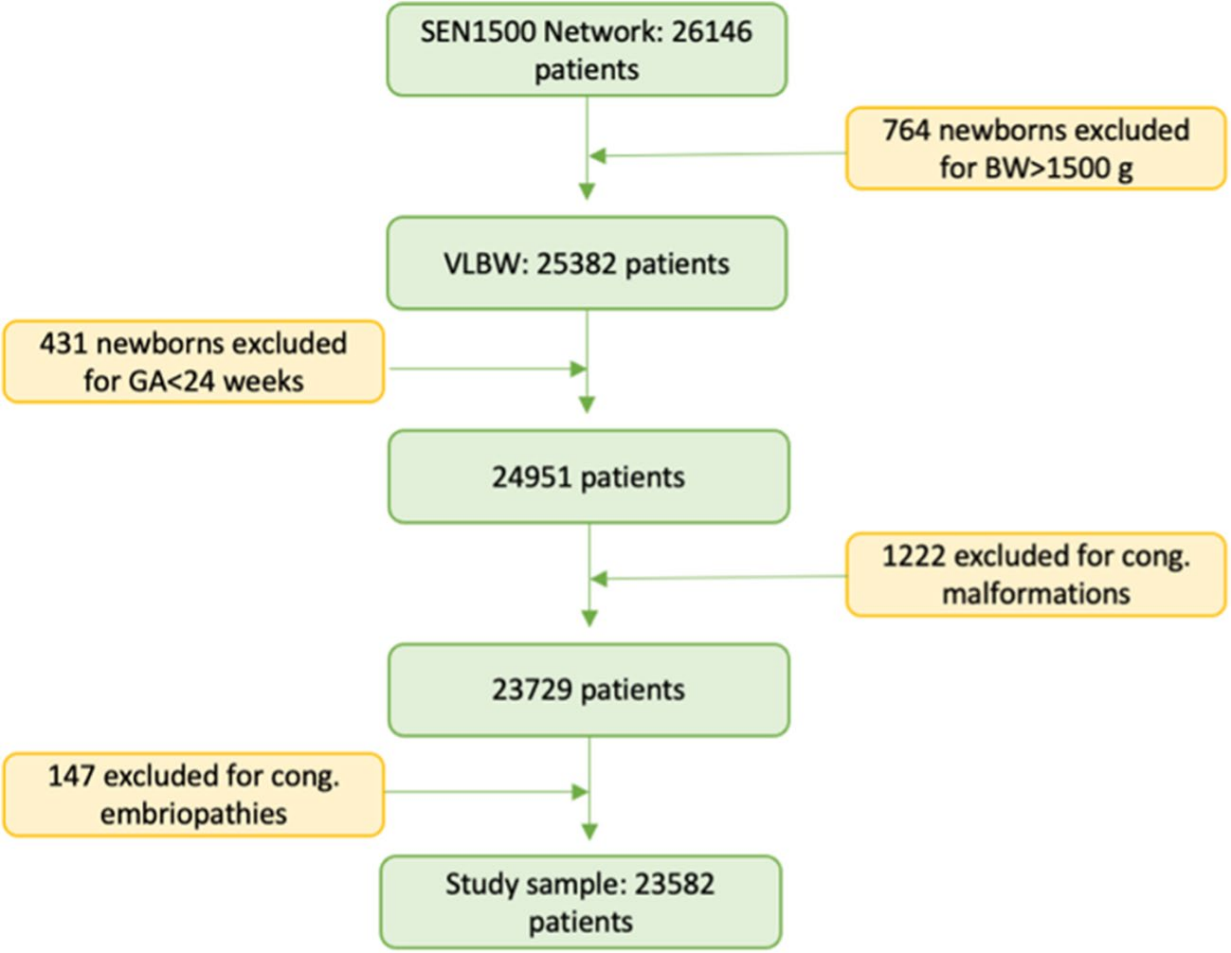
Flowchart of the population included in the study

The initial data from SEN1500 included 26,146 very preterm infants or very low weight for gestational age. From this sample, a total of 25,382 very-low-weight gestational-age infants were eligible for the study. In total, 2565 patients met the exclusion criteria due to the following: major congenital anomalies (1222 patients), congenital embryopathies with growth impairment (147 patients), gestational age less than 24 weeks of gestation (431 patients). The final study sample was constituted by 23,582 patients as represented in the flowchart in Fig. 1.

### Characteristics of the study population

Patients included were admitted from January 2011 to December 2020 in 77 participating sites. Median gestational age at birth was 29 (interquartile range [IQR] 4) weeks, and 50.4% of the sample were males (11,859 infants). Perinatal characteristics of the population included are summarized in Table 1.

**Table 1:** Perinatal characteristics of the study population

	Median (IQR) for quantitative data and absolute number (percentage) for qualitative data
Gestational age at birth (weeks)	29 (4)
Weight at birth (g)	1119 (460)
Length at birth (cm)	37.15 (5)
Head circumference at birth (cm)	26.3 (3.5)
Male gender	11,859 (50.3)
Prenatal corticosteroids	No prenatal steroids 2568 (10.9) Partial course 4253 (18) Complete course 16,575 (70.3) Unknown course 186 (0.7)
Multiple gestations	2807 (11.9) Monochorionic 710 (3) Bichorionic 2097 (8.9)
IVF	4705 (19.9)
Mode of delivery with cesarean section	17,304 (73.4)
APGAR score 5 min less than 5	968 (4.1)
Crib 1 score	6.86 (4.8)
Extramural deliveries	1054 (4.5)
Resuscitation at delivery	Intubation 6515 (27.6) Chest compressions 1225 (5.2) Epinephrine 742 (3.1)

Median weight, length, and head circumference at birth were 1119 (IQR 460) grams, 37.15 (IQR 5) centimeters, and 26.3 (IQR 3.5) centimeters, respectively. Classification of growth patterns at birth by weight and growth curve are presented in Table 2. Anthropometric measurements at the time of discharge were median weight 2200 (IQR 565) grams, length 44.5 (IQR 3.5) centimeters, and head circumference 32.5 (IQR 2) cm.

**Table 2:** Prevalence of growth patterns by weight at birth with the different growth curves studied

	Fenton	Olsen	INTERGROWTH-21st
Small for gestational age	6501 (27.6%)	6275 (26.6%)	7338 (31.1%)
Appropriate for gestational age	16,586 (70.3%)	15,279 (64.8%)	15,833 (67.1%)
Large for gestational age	495 (2.1%)	2028 (8.6%)	411 (1.7%)

Global survival in the sample was 89.4% as 2499 patients died during the hospitalization in the NICU. From the patients in whom information was available, overall survival without morbidities was 49% (10,000 patients). Morbidities experienced during admission are summarized in Table 3. Prevalence of morbidities and mortality during admission of infants classified as SGA by weight with the different growth curves is summarized in Table 4.

**Table 3:** Morbidities and mortality of the study population

	VLBW (percentage)
Mechanical ventilation	9979 (42.3%)
Pneumothorax	918 (3.9%)
Anemia requiring transfusion	8837 (37.5%)
Necrotizing enterocolitis	1459 (6.2%)
Patent ductus arteriosus	6537 (27.7%)
Shock requiring cardioactives/vasopressors	4811 (20.4%)
Acute kidney injury	1507 (6.4%)
ROP more than 2 stage	688 (2.9%)
Bronchopulmonary dysplasia	5200 (22%)
Periventricular leukomalacia	2939 (12.5%)
Intraventricular hemorrhage grades 3–4	1840 (7.8%)
Mortality	2382 (10.1%)

**Table 4:** Morbidities and mortality of the study population classified as SGA at birth by weight

	SGA by Fenton (6501 patients)	SGA by Olsen (6275 patients)	SGA by INTERGROWTH-21st (7338 patients)
Mechanical ventilation	1518 (23.4%)	1526 (24.3%)	1821 (24.8%)
Pneumothorax	137 (2.1%)	129 (2.1%)	155 (2.1%)
Anemia requiring transfusion	1630 (25.1%)	1656 (26.4%)	1931 (26.3%)
Necrotizing enterocolitis	347 (5.3%)	340 (5.4%)	394 (5.4%)
Patent ductus arteriosus	831 (12.8%)	837 (13.3%)	1028 (14%)
Shock requiring cardioactives/vasopressors	867 (13.4%)	861 (13.7%)	1002 (13.7%)
Acute kidney injury	295 (4.6%)	298 (4.8%)	314 (4.3%)
ROP more than 2 stage	121 (1.9%)	120 (1.9%)	130 (1.8%)
Bronchopulmonary dysplasia	78 (1.2%)	83 (1.3%)	87 (1.2%)
Periventricular leukomalacia	479 (7.4%)	467 (7.4%)	538 (7.3%)
Intraventricular hemorrhage grades 3–4	590 (9.1%)	237 (3.8%)	262 (3.6%)
Mortality	550 (8.5%)	539 (8.6%)	572 (7.8%)

### Prevalence of EUGR

**Fig. 2:**
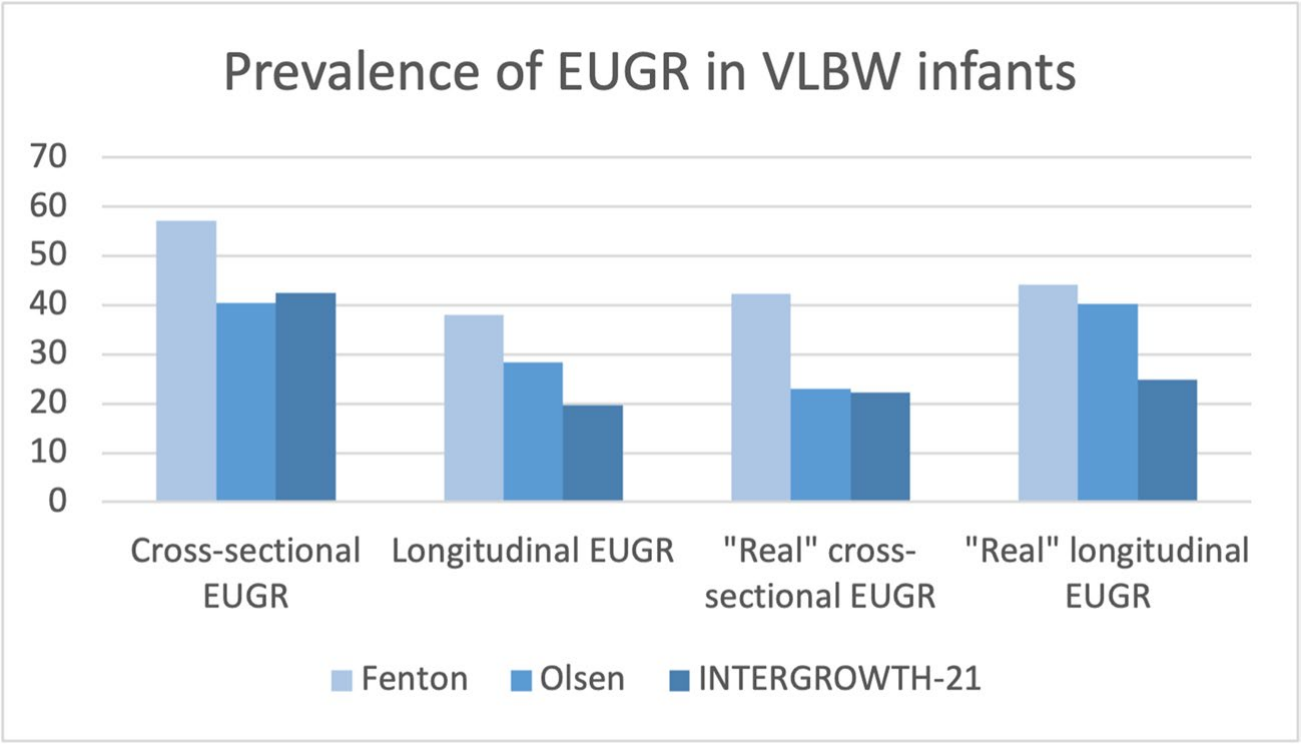
Prevalence of EUGR (weight) by growth reference in the study sample of VLBW infants

The prevalence of EUGR with cross-sectional, longitudinal, and “true” criteria for weight in the study sample varied with the use of different references as depicted in Table 5. Overall, the prevalence of EUGR is higher in all the proposed definitions of the concept with the use of Fenton reference in our population as presented in Fig. 2. This tendency persisted by sex and evaluating length and head circumference, as described in Table 6.

**Table 5:** Prevalence of EUGR by weight with the different criteria proposed

	Fenton	Olsen	INTERGROWTH-21st
Cross-sectional EUGR			
VLBW	13,341 (56.6%)	8862 (37.6%)	9541 (40.4%)
Men	6655 (56.1%)	4500 (37.9%)	5259 (44.3%)
Women	6686 (57.0%)	4362 (37.2%)	4282 (36.5%)
Longitudinal EUGR			
VLBW	8972 (38%)	6700 (28.4%)	4630 (19.6%)
Men	4430 (37.4%)	3333 (28.1%)	2903 (24.5%)
Women	4542 (38.7%)	3367 (28.7%)	1727 (14.7%)
“True” cross-sectional EUGR			
VLBW	7147 of 16,890 (42.3%)	3753 of 16,240 (23.1%)	3412 of 15,276 (22.3%)
Men	3514 of 8429 (41.7%)	1866 of 8028 (23.2%)	2072 of 7647 (27.1%)
Women	3633 of 8461 (42.9%)	1887 of 8212 (23%)	1340 of 7629 (17.6%)
“True” longitudinal EUGR			
VLBW	7456 of 16,890 (44.1%)	6526 of 16,240 (40.2%)	3811 of 15,276 (24.9%)
Men	3684 of 8429 (43.7%)	3237 of 8028 (40.3%)	2331 of 7647 (30.5%)
Women	3772 of 8461 (44.6%)	3289 of 8212 (40.1%)	1480 of 7629 (19.4%)

**Table 6:** Prevalence of EUGR by length and head circumference with the different criteria proposed

	Fenton	Olsen	INTERGROWTH-21st
Length			
Cross-sectional EUGR	11,487 (48.7%)	9001 (38.1%)	10,926 (46.3%)
Longitudinal EUGR	9713 (41.2%)	8450 (36.2%)	7662 (32.5%)
“True” cross-sectional EUGR	6645 of 14,829 (44.8%)	5497 of 14,923 (36.8%)	5466 of 13,440 (40.7%)
“True” longitudinal EUGR	8336 of 14,829 (56.2%)	7941 of 14,923 (53.2%)	5564 of 13,440 (41.4%)
Head circumference			
Cross-sectional EUGR	5368 (22.7%)	2448 (10.4%)	6556 (27.8%)
Longitudinal EUGR	4173 (17.7%)	4053 (17.2%)	4100 (17.4%)
“True” cross-sectional EUGR	2649 of 15,168 (17.5%)	1302 of 15,629 (8.3%)	3262 of 14,345 (22.7%)
“True” longitudinal EUGR	3921 of 15,168 (25.9%)	3992 of 15,628 (25.5%)	3473 of 14,345 (24.2%)

The prevalence of EUGR by growth pattern at birth (weight), classified by SGA, AGA, and LGA, was calculated with the different criteria and growth curves by weight and is presented in Table 7.

**Table 7:** Prevalence of EUGR growth pattern at birth with the different growth curves studied

	Fenton	Olsen	INTERGROWTH-21st
Small for gestational age			
Cross-sectional EUGR	6194 (95.3%)	5109 (81.4%)	6129 (83.5%)
Longitudinal EUGR	1516 (23.3%)	174 (2.8%)	819 (11.2%)
Adequate for gestational age			
Cross-sectional EUGR	7092 (42.7%)	3643 (23.8%)	3390 (21.4%)
Longitudinal EUGR	7071 (42.6%)	4980 (32.6%)	3640 (23%)
Large for gestational age			
Cross-sectional EUGR	55 (11.1%)	110 (5.4%)	22 (5.4)
Longitudinal EUGR	385 (77.8%)	1546 (76.2%)	171 (41.6%)

### Concordance among Fenton, Olsen, and INTERGROWTH-21st references for diagnosis of EUGR

Fleiss’ kappa coefficients and 95% confidence intervals for diagnosis of extrauterine growth restriction using the three assessed growth charts by each definition of EUGR are depicted in Table 8. Agreement amongst charts was good for cross-sectional EUGR criteria by weight and moderate for the rest of the criteria of EUGR by weight (longitudinal, “true” cross-sectional, and “true” longitudinal) in VLBW infants. Concordance for EUGR criteria by length and head circumference was good or very good with all the definitions studied.

**Table 8:** Fleiss’ kappa coefficients and 95% confidence intervals for each definition amongst Fenton, Olsen, and INTERGROWTH-21st growth charts

	Fleiss’ kappa	95% CI
Weight		
Cross-sectional EUGR	0.729	(0.721, 0.737)
Longitudinal EUGR	0.568	(0.560, 0.576)
“True” cross-sectional EUGR	0.612	(0.602, 0.621)
“True” longitudinal EUGR	0.584	(0.580, 0.599)
Length		
Cross-sectional EUGR	0.867	(0.859, 0.875)
Longitudinal EUGR	0.698	(0.689, 0.706)
“True” cross-sectional EUGR	0.840	(0.829, 0.850)
“True” longitudinal EUGR	0.678	(0.668, 0.689)
Head circumference		
Cross-sectional EUGR	0.633	(0.625, 0.641)
Longitudinal EUGR	0.726	(0.718, 0.734)
“True” cross-sectional EUGR	0.603	(0.594, 0.613)
“True” longitudinal EUGR	0.731	(0.722, 0.741)

## Discussion

There is no consensus definition of extrauterine growth restriction nor ideal growth reference for the assessment. In consequence, there is significant variability in the prevalence of EUGR depending on the definition and growth chart used as reflected in this study. In our population, the prevalence ranged from 19.6 using the EUGR longitudinal definition to 56.6% using the cross-sectional definition.

According to the cross-sectional definition (weight at discharge-discharge below the 10th percentile for GA), EUGR prevalence was reported to be 56.8% in Shanghai [32], 50.3% by Vermont Oxford Network [33], 74% in newborns with GA less than 30 weeks in Italy [6], and 59.2% in a Spanish cohort [34].

For the longitudinal definition, studies also differ in the choice of cutoff for the change in *z*-score, which results in difficulties in interpreting and comparing findings. Peila et al. [6] reported a prevalence of 92% of longitudinal EUGR defined as the loss of 1 standard deviation (SD) in preterms < 30 weeks while Franz et al. [28] described 50% in preterms < 26 weeks of GA. For longitudinal EUGR described as a change in more than 2 *z*-score from birth to discharge, a study from Israel reported a prevalence ranging from 11.7 to 7.2% for severe EUGR [35].

The most recent criteria proposed is “true” EUGR. Prevalence of the newest EUGR concept, which would include cross-sectional and longitudinal criteria in non-SGA infants at birth, is also variable. “True” cross-sectional EUGR prevalence has previously been described as 60.4% [6], 12.3% [10], and 17% (in non-SGA babies with birthweight between 750 and 1250 g) [36]. “True” EUGR with longitudinal criteria has been estimated from 39.6 to 52.7% with 1 SD cutoff [37] and 13% using 2 SD criteria [38].

As proposed by Peila et al. [6], the different definitions measure different aspects of the growth. While the cross-sectional criteria only consider the evaluation at discharge, the longitudinal criteria evaluate changes from birth to discharge. Moreover, “true” EUGR would consider only those infants with postnatal growth impairment, excluding those with fetal growth restriction [10].

Moreover, variability in prevalence persists amongst the three growth charts studied when considering each definition alone. Overall, prevalence is higher in our study with the use of the Fenton growth chart and lower with Olsen (cross-sectional and “true” cross-sectional) and IW-21 (longitudinal and “true” longitudinal). El Rafei et al. [39], Reddy et al. [40], González-García et al. [37], and Tuzun et al. [41] reported similar results, with higher prevalence of EUGR with cross-sectional definition using Fenton charts than with intergrowth-21st charts. In contrast, Lan et al. [42] did not find significant differences in the incidences of cross-sectional EUGR by Fenton and Intergrowth-21st charts. Despite our choice of criteria for the cross-sectional EUGR definition, it is important to highlight the prevalence described in studies using other criteria for this definition. A study by Yazidi et al. [7] described a significantly higher prevalence of cross-sectional EUGR (using the criteria of weight at discharge less than − 2 *z*-score) with Fenton curves than INTERGROWTH-21st. Our study describes results consistent with the literature for the longitudinal EUGR, with higher prevalence when using Fenton charts compared to Intergrowth-21st [42, 43]. It is important to consider for the interpretation of the previously described results that the varying methodology followed for the construction of each growth curve could potentially also affect these results. While Fenton charts from 2013 are based on the study of the anthropometry of 3,986,456 infants and Olsen studied 391,681 preterms, INTERGROWTH-21st preterm growth standards include a smaller number of preterm patients and are based on the study of less than 500 patients. Consideration should also be given to the prospective study conducted in INTERGROWTH-21st compared to Fenton and Olsen (retrospective).

Agreement among the three assessed growth charts for each definition of EUGR was good or very good for EUGR criteria by length and head circumference. EUGR by weight, which has been traditionally used for the definition, has good concordance only for the cross-sectional EUGR criteria and is moderate for the rest of the criteria of EUGR by weight (longitudinal, “true” cross-sectional, and “true” longitudinal) in VLBW infants. Results are consistent with those from González-García et al. [37]. In contrast, a study from Lan et al. [42] reported low concordances for cross-sectional EUGR for weight, length, and head circumference with Fenton and Intergrowth-21st in infants < 32 weeks of GA, with good concordance between those previously described for longitudinal EUGR. Previous studies have also reported low agreement between the cross-sectional and longitudinal definitions of EUGR with Cohen’s kappa coefficient from 0.07 to 0.38 [6]. Moreover, a recent study from 2024 has evaluated the same growth curves used in our study (Fenton, Olsen, and INTERGROWTH-21st) and studied the discrimination power of the curves for the risk of mortality in different populations, finding that INTERGROWTH-21st fetal growth curves had the strongest discrimination power for very-low-birthweight infants, the population assessed in our study [44].

It is important to consider that INTERGROWTH-21st preterm postnatal growth standards present some particularities for the anthropometric evaluation of preterm infants. While length and head circumference can be monitored with this standard from birth, as physiological weight loss was not considered for their development, weight centiles may be assigned and evaluated when preterms start regaining weight.

Considering all the above presented, it is crucial to be mindful of the significant variability of diagnosis depending on the choice of growth chart and definition of EUGR as establishing the diagnosis of EUGR will likely have a direct impact on the clinical evaluation and follow-up of the patients.

Limitations of the study include that our evaluation of EUGR in very-low-birthweight infants was conducted with the exclusion of infants born at less than 24 weeks of gestational age. This exclusion was performed due to the absence of percentiles and *z*-score for anthropometry at this gestational age with INTERGROWTH-21st, to allow comparisons among the three studied growth curves, but it may have impacted the results obtained. Moreover, the retrospective nature of the study conducted with limited data on nutrition practices did not allow us to study any potential association between changes in nutritional practices and EUGR. Another limitation of our study is that no follow-up after discharge from the hospital was included, so morbidities and mortality studied are limited to in-hospital. Finally, despite the study being multicenter and the size of the sample included, our study includes only infants cared for at hospitals participating in SEN1500, which can also limit the external validity of the study.

## Conclusions

Prevalence of EUGR varied in our cohort of VLBW infants with the most commonly used definitions of the term (cross-sectional, longitudinal, “true” cross-sectional, and “true” longitudinal) using Fenton, Olsen, and Intergrowth-21st. Moreover, agreement among the three growth charts was moderate for all the definitions of EUGR by weight except cross-sectional and good or very good for length and head circumference. The choice of reference chart can impact the establishment of the diagnosis of EUGR.

## Data Availability

All data, code, and material will be available on request following publication.

## Abbreviations

cm: Centimeters
EUGR: Extrauterine growth restriction
g: Grams
HC: Head circumference
IQR: Interquartilic range
L: Length
NICU: Neonatal intensive care unit
SD: Standard deviation
SGA: Small for gestational age
VLBW: Very low birthweight
W: Weight
